# Determinants of Life Satisfaction and Mental Wellbeing in the Danish General Population: Shared and Distinct Associations

**DOI:** 10.3389/ijph.2025.1608531

**Published:** 2025-09-12

**Authors:** Mette Rasmussen, Ziggi Ivan Santini, Mohsen Joshanloo, Christina Bjørk Petersen, Anne Illemann Christensen

**Affiliations:** ^1^ National Institute of Public Health, University of Southern Denmark, Copenhagen, Denmark; ^2^ Copenhagen Research Unit for Recovery, Mental Health Services, Mental Health Centre Amager, Copenhagen, Denmark; ^3^ Department of Psychology, Keimyung University, Daegu, Republic of Korea; ^4^ Center for Clinical Research and Prevention, Copenhagen University Hospital, Copenhagen, Denmark

**Keywords:** life satisfaction, mental wellbeing, cross-sectional survey, health measures, correlation

## Abstract

**Objective:**

To examine the correlation between two wellbeing components: life satisfaction and mental wellbeing and identify key determinants (sociodemographic, health, behavioural, social).

**Methods:**

This cross-sectional study used data from 10,196 adults from the nationally representative Danish National Health Survey 2023. Main outcomes were self-reported life satisfaction measured on a single-item scale from 0–10 and mental wellbeing by the short version of the Warwick-Edinburgh Mental Wellbeing Scale (SWEMWBS). Correlations were examined using Spearman’s rho (ρ) and Pearson’s correlation coefficient (r). Linear regression models estimated associations between key determinants and the outcomes.

**Results:**

The two outcomes were strongly correlated, yet remained distinct. Wellbeing scores were similar across sex, age, ethnicity, education, employment, BMI, tobacco, and alcohol use. Scores varied by marital status, financial strain, self-rated health, chronic illness, physical activity, loneliness, stress, anxiety, and depression risk. Associations with pain, sleep quality, social support, and leisure activities were mixed.

**Conclusion:**

Despite a strong correlation discriminant validity was maintained. Wellbeing outcomes should not be treated as interchangeable, as their associations vary across population groups.

## Introduction

Wellbeing has been the subject of increasing attention as a key public health issue during the past decade [1, 2]. Wellbeing is a comprehensive multidimensional concept that encompasses various aspects such as evaluative, emotional and cognitive, as well as aspects pertaining to social relationships, financial stability, and a sense of meaning and purpose in life. It covers physical, mental, and social wellbeing, extending beyond the presence/absence of ill-being (illness or disorder), to a state of wellness, where individuals e.g., feel satisfied, optimistic, energetic, healthy, and capable of managing daily challenges [3].

Integrating positive health measures into public health and epidemiological practice represents a strategic shift from a reactive model — focused on disease prevention and treatment — to a proactive model that fosters overall wellbeing. By emphasizing factors such as emotional resilience, social connectedness, physical vitality, and a sense of purpose, positive health approaches contribute to healthier, more engaged populations. These measures not only improve individual quality of life but also reduce the incidence and burden of chronic diseases, thereby lowering healthcare costs and enhancing system sustainability. From an epidemiological perspective, tracking and promoting positive health indicators allows for a more nuanced understanding of population health dynamics, supports early intervention strategies, and strengthens public health responses to emerging challenges. Moreover, positive health frameworks promote equity by addressing upstream determinants and empowering communities to thrive.

To disentangle these dimensions, wellbeing is often operationalized using different measures, each capturing unique aspects of individuals’ lived experiences. Two components of wellbeing are the focus of this study: life satisfaction and mental wellbeing. The two are cross-sectionally and temporally interconnected, highlighting the bidirectional relationship between them [2].

Life satisfaction refers to how a person evaluates their life as a whole — a cognitive, overall assessment of how satisfied they are with their life. It is often considered as the evaluative component of subjective wellbeing [4], and is of interest in public health research due to its broad utility and implications for health [5]. It is influenced by factors such as physical activity, sleep, smoking, and alcohol consumption [6, 7]. High life satisfaction is both an outcome and a predictor of good health, associated with lower mortality [5, 8, 9]. It is typically measured by asking individuals to evaluate their overall satisfaction with life, based on personal criteria and reflective judgment. The measure is intentionally broad, allowing respondents to subjectively weigh aspects of life they consider important [10].

Mental wellbeing, in contrast, is a broader concept that includes both positive emotions and psychological functioning. It incorporates both hedonic (feeling good) and eudaimonic (functioning well) facets. It is characterized by positive emotions, positive cognitive-psychological and social functioning, and a sense of purpose [10], enabling individuals to cope with stress, achieve goals, and contribute to their communities [3]. Higher levels of mental wellbeing reduce the risk of common mental disorders and predict lower healthcare utilization and loss of productivity [11–13]. Unlike life satisfaction, which reflects an overall assessment of life, mental wellbeing captures ongoing psychological and social functioning, making it particularly relevant for understanding health and resilience over time.

From a public health perspective, understanding the implications of different wellbeing constructs, as well as associations with other health constructs are crucial, as it can be used to understand how individuals perceive their lives and identify factors that contribute to improving both psychological and somatic health outcomes.

To date, research has provided valuable insights into the factors influencing wellbeing, including physical health, socio-economic status, social relationships, risk and health behaviour. Further changes in health behaviours have been linked to mental health and life satisfaction [5]. Chen et al., for example, found that changes in physical and sedentary behaviours during the COVID-19 pandemic impacted mental wellbeing and life satisfaction [7]. Furthermore, Christensen et al. revealed that mental health status predicted several social life events, such as educational attainment, employment status, marital status, and parenthood [5]. Yet, high income does not necessarily enhance mental wellbeing, while it improves life satisfaction [5].

Given that different wellbeing measures (e.g., life satisfaction and mental wellbeing) may be sensitive to different factors, it is essential to understand how associations with socio-demographic and health-related factors differ. Otherwise, wellbeing measures may be incorrectly assumed to reflect identical constructs influenced by the same predictors. By comparing how selected socio-demographic and health determinants predict evaluative and mental wellbeing outcomes, this study aims to identify similarities and differences to close some of the remaining gaps in our understanding of the relationship between life satisfaction and mental wellbeing, as well as their associations with health behaviours. This nuanced perspective may contribute to more targeted and effective public health interventions aimed at improving both life satisfaction and mental wellbeing in the Danish general population. The Danish National Health Survey from 2023 (DNHS-2023) provides a valuable resource for examining these associations in a Danish context [6].

### Objective

The aim of this study was to examine the correlation between life satisfaction and mental wellbeing and identify key determinants for both constructs, including health status, socio-economic position, social relationships, and health-related behaviours. By elucidating these associations, the study aimed to generate evidence that can inform targeted public health policies and interventions to enhance mental wellbeing and overall life satisfaction.

## Methods

### Study Design and Data Collection

This study was based on data from DNHS-2023 which is a nationally representative cross-sectional health survey. A random sample of 25,000 residents in Denmark aged 16 years or older were invited to participate. The sample was drawn from the Danish Civil Registration System [14] where all citizens with official residence in Denmark are registered by a unique personal identification number. Data were collected through self-administered questionnaires (paper/web), either in paper or web format. A majority of 96.6% responded via the web-based version, while 3.4% of the participants completed the paper questionnaire. The survey design has been described in detail elsewhere [15].

The study was reviewed and approved by the Scientific Committee of the University of Southern Denmark and was registered with the university’s research register (approval number: 11.702). All procedures adhered to the EU GDPR guidelines, ensuring participants’ data security and confidentiality.

### Outcomes

In this study life satisfaction and mental wellbeing were the two main outcomes. Both outcomes were self-reported.

Life satisfaction was measured by the survey question: *“Overall, how satisfied are you with your life nowadays?”,* where participants were asked to respond on a scale from 0–10, where 0 means extremely dissatisfied and 10 means extremely satisfied [16, 17].

Mental wellbeing was measured by the short version of the Warwick-Edinburgh Mental Wellbeing Scale (SWEMWBS) [18], which consists of seven questions asking the participants to rate their thoughts and feelings over the past 2 weeks. This scale focuses on aspects of mental functioning using positively worded statements. The included items assess optimism, self-efficacy, relaxation, ability to solve problems, cognitive clarity, social connection, and autonomy.

Each question was rated on a 5-point Likert scale from “at no time” (1 point) to “all the time” (5 points), with higher scores indicating better mental wellbeing. The sum of the responses was converted into an overall scale score of 7–35 points using a conversion table [19]. The scale has been validated for use in the Danish population [20]. To make the results for life satisfaction and mental wellbeing easier to compare, the SWEMWBS scores were converted to a 0–10 scale (SWEMWBS_10_) using the formula:

$${SWEMWBS}_{10}=\frac{\left(SWEMWBS-7\right)*10}{\left(35-7\right)}$$

The validation literature regarding single-item measures is limited, but even though single-item life satisfaction questions lack the depth of multi-item scales, several studies suggest that they still show acceptable reliability and validity for use in population-level research [21–23]. Mental wellbeing is supported by robust psychometric evidence. The widely used instrument WEMWBS exhibits high internal consistency, construct validity, and cross-cultural applicability. The measure enables reliable assessment of positive health outcomes across diverse populations and contexts [24, 25].

### Other Variables

We mainly used data from the DNHS-2023, but some of the variables were also derived from the Danish Civil Registration System [14] as part of the sampling frame. All information was collected as categorical variables, except age and body mass index (BMI) which were collected as continuous variables but categorised before analysis. The variables were sub-categorised and presented in Table 1, along with their operationalisation.

**TABLE 1:** Presentation of variables used in the study and how they were categorised and labelled (The Danish National Health Survey, Denmark, 2023).

Variable	Categorisation
Sociodemographic factors	
Sex (binary)	Men/Women
Age	16–24/25–44/45–64/65+ years
Ethnic background [15]	Danish/Western/Non-Western
Marital status [15]	Married or cohabiting/Divorced or separated/Widowed/Unmarried
Education	Basic school/Upper secondary or vocational school/Higher education/Missing
Employment	Employed/Unemployed/Pensioners or early retirement/Other/Missing
Difficulty paying bills	Never/Some few months/∼ Half of the months in a year/Every month
Health factors	
Self-rated health (dichotomised)	Good health (excellent, very good, good)/Less than good health (less good, poor)
Longstanding illness [15]	No/Yes
Frequency of physical pain	Never/Some days/Most days/Every day
BMI (body mass index)	Less than 18.5/18.5 to <25/25 to <30/30 or greater
Cohen’s Perceived Stress Scale [26, 27]	Medium/Low stress level (<18)/High stress level (≥18)
Generalized Anxiety Disorder 2-item (GAD-2) [28]	Low risk of anxiety (<3)/High risk of anxiety (≥3)
Patient Health Questionnaire-2 (PHQ-2) [29]	Low risk of depression (<3)/High risk of depression (≥3)
Health behaviour factors	
Physical activity in leisure time (Saltin-Grimby) [30]	Sedentary activity/Light activity/High/Moderate activity
Enough sleep to feel rested	Yes, usually/Yes, but not often enough/No, never (almost never)
Smoking status	Never smoker/Ex-smoker/Occasional smoker/Daily smoker
Alcohol consumption	None/<21 (men) or <14 (women) standard drinks week/≥21 (men) or ≥14 (women) standard drinks week
Social relations factors	
Loneliness [31]	Not lonely/Lonely
Social support	Yes, always/Yes, mostly/Yes, sometimes/No, never or almost never
Leisure activities with others	Several times a week/App. once a week/1–3 times per month/Less than once per month/Never

### Statistical Analyses

Categorical data were reported in absolute numbers and percentages. The main outcomes of life satisfaction and mental wellbeing were reported using median and range, due to their robustness against outliers and non-normal data distributions.

To reduce the impact of non-response calibrated weights were constructed by Statistics Denmark and applied to the data [32, 33]. The weights were based on the following information from administrative registers: sex, age, highest completed level of education, income, socioeconomic grouping, family type, ethnic background, homeowner/tenant status, and number of visits to the general practitioner and hospital contacts in the year 2021 [15]. In the analyses, those who were less likely to participate were given a higher weight to represent the larger number of non-respondents with similar characteristics. Likewise, those more likely to participate were given a lower weight.

Correlations between life satisfaction and mental wellbeing were examined by visualising both parametric and non-parametric values, and by calculation of Spearman’s rho correlation coefficient (ρ) and Pearson’s correlation coefficient (r) to assess monotonic and linear correlation, respectively. Correlation coefficients were interpreted as very weak if < 0.20, weak if between 0.2 and <0.40, moderate if between 0.40 and <0.60, strong if between 0.60 and <0.80 or very strong if ≥0.80 [34].

Linear regression models were used to estimate the association between different sociodemographic or health measures and each of the two main outcomes separately. All predictors were analysed using a model adjusted for age and sex, incorporating weights to account for the sampling design. Results were reported as regression coefficients and 95% confidence intervals (95% CIs). Multicollinearity was assessed using the variance inflation factor (VIF). In the employment model, the VIF for employment status (5.43 for life satisfaction and 7.09 for mental wellbeing) suggests a potential issue of multicollinearity with age, warranting further investigation. Although removing age reduced multicollinearity, it led to substantial changes in the estimated association between employment and the outcomes, suggesting that age is a key confounder. Therefore, age was retained in the model to ensure valid inference and consistency across models.

Analyses were based on respondents with full data only, using a complete case approach. All analyses were performed using R version 4.4.1 (R Foundation for Statistical Computing) [35], and the packages *ggplot2* and *ggforestplot* for graphs. In all statistical tests a two-sided p-value of <0.05 was considered statistically significant.

## Results

A total of 10,196 adults participated in the DNHS-2023. The characteristics of the study population are presented in Table 2. This table also shows the scores on life satisfaction and mental wellbeing according to the selected sociodemographic factors.

**TABLE 2:** Number and percentages of participants, and levels of life satisfaction and mental wellbeing according to sociodemographic factors (The Danish National Health Survey, Denmark, 2023).

Sociodemographic factor		n	(%)	Life satisfaction (0–10)	Wellbeing (0–10) SWEMWBS converted
		No weights applied		Weighted median (min-max)	Weighted median (min-max)
Men		4,481	(43.9)	7.5 (0–10)	6.0 (0–10)
	16–24 years	393	(3.9)	6.5 (0–10)	5.6 (0–10)
	25–44 years	847	(8.3)	6.5 (0–10)	5.6 (0–10)
	45–64 years	1,538	(15.1)	7.5 (0–10)	6.0 (0–10)
	65 years or older	1,703	(16.7)	7.5 (0–10)	6.3 (0–10)
Women		5,715	(56.1)	7.5 (0–10)	5.6 (0–10)
	16–24 years	520	(5.1)	6.5 (0–10)	5.0 (0–10)
	25–44 years	1,273	(12.5)	6.5 (0–10)	5.3 (1.5–10)
	45–64 years	1,979	(19.4)	7.5 (0–10)	6.0 (0–10)
	65 years or older	1,943	(19.1)	7.5 (0–10)	6.3 (0–10)
Education (highest level)					
	Basic school	849	(8.3)	7.5 (0–10)	5.6 (0–10)
	Upper secondary or vocational school	2,769	(27.2)	7.5 (0–10)	6.0 (0–10)
	Higher education	4,469	(43.8)	7.5 (0–10)	6.0 (0–10)
	Missing	2,109	(20.7)	6.5 (0–10)	5.3 (0–10)
Employment status					
	Employed	4,370	(42.9)	7.5 (0–10)	6.0 (0–10)
	Unemployed	192	(1.9)	6.5 (0–10)	4.8 (0–10)
	Pensioner/early retiree	4,274	(41.9)	7.5 (0–10)	6.0 (0–10)
	Other	1,171	(11.4)	6.5 (0–10)	5.3 (0–10)
	Missing	189	(1.9)	6.5 (0–10)	5.1 (3.0–9.1)
Marital status					
	Married/cohabiting	5,576	(54.7)	7.5 (0–10)	6.0 (0–10)
	Divorced/separated	1,180	(11.6)	7.5 (0–10)	6.0 (0–10)
	Widowed	699	(6.9)	7.5 (0–10)	6.3 (0–10)
	Unmarried	2,741	(26.9)	6.5 (0–10)	5.3 (0–10)
Total		10,196	(100)	7.5 (0–10)	5.95 (0–10)

Overall, the study population consisted of more women (56.1%) than men and most participants were 45 years or older (70.3%). Most participants had a higher education (43.8%) and were either employed (42.9%) or not on the labour market due to retirement (41.9%).

While men and women had the same median life satisfaction, men reported slightly higher mental wellbeing compared to women. Both variables increased with age. Life satisfaction was consistent across different educational levels, but mental wellbeing was lower for those with only a basic school education. The highest levels of life satisfaction and mental wellbeing were observed in employed and retired individuals, whereas the unemployed had lower levels in both measures, particularly in mental wellbeing. Married/cohabiting, divorced, and widowed participants reported higher life satisfaction and mental wellbeing compared to unmarried participants.

### Correlation

**FIGURE 1:**
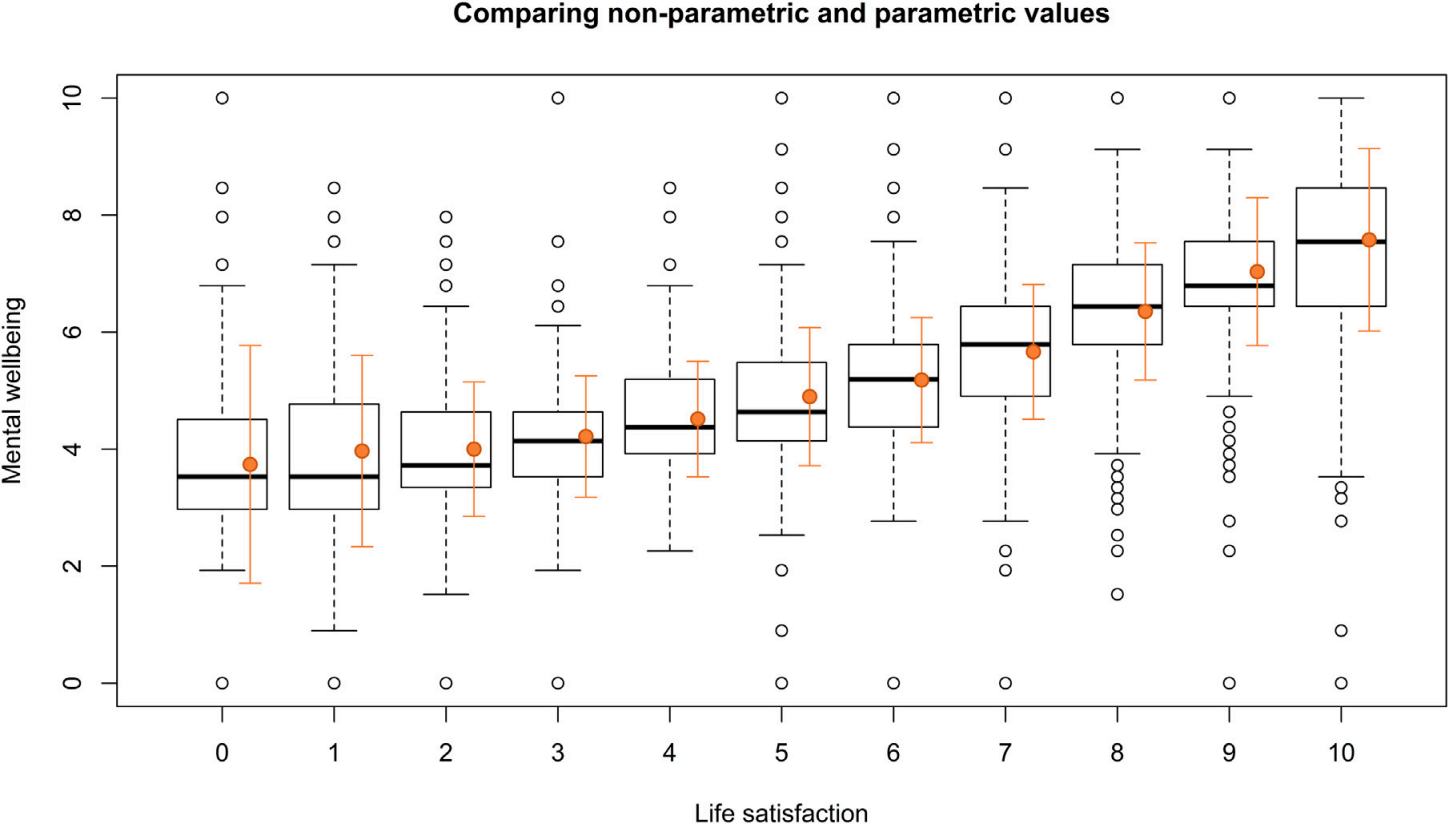
Comparison of non-parametric (black boxplots) and parametric (orange dots) analyses of the correlation between life satisfaction and mental wellbeing (SWEMWBS_10_). The boxplots show the median and interquartile range, with outliers represented as individual points. The parametric approach is displayed using the mean and standard deviation (SD) (The Danish National Health Survey, Denmark, 2023).

The visual inspection of the parametric and non-parametric plots illustrating the strength of the relationship between life satisfaction and mental wellbeing (Figure 1) revealed that the central tendency measures were quite similar, suggesting that the distributions of mental wellbeing for each life satisfaction value were approximately normal. However, while the parametric approach resulted in a smoother general trend, the non-parametric approach highlighted the variability and outliers in the data.

Calculations of Spearman’s rho (ρ = 0.64, p < 0.0001) and Pearson’s correlation coefficient (r = 0.62 [95% CI: 0.60; 0.63], p < 0.0001) were rather similar and established a strong correlation between life satisfaction and mental wellbeing [34]. According to the linear correlation 38% of the changes in one outcome could be explained by changes in the other (R^2^ = 0.38). The results indicate a strong and consistent positive linear relationship between life satisfaction and mental wellbeing, and the correlation was robust to the model assumptions.

### Determinants of Life Satisfaction and Mental Wellbeing

#### Sociodemographic Factors

**FIGURE 2:**
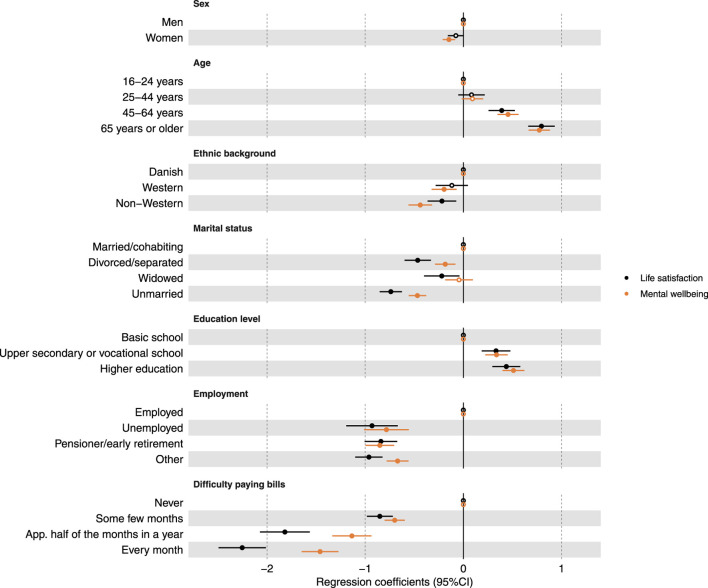
Presentation of associations (regression coefficients and 95% confidence interval) between life satisfaction or mental wellbeing and sociodemographic factors. All analyses are adjusted for sex and age and incorporating weights to account for the sampling design. Closed circles represent associations with a p-value <0.05, while open dots indicate associations with a p-value ≥0.05 (The Danish National Health Survey, Denmark, 2023).

Figure 2 shows the results of the linear regression analyses, highlighting associations between sociodemographic factors and evaluative and mental wellbeing. Women had slightly lower regression coefficients than men, especially for mental wellbeing. Age showed a positive association, with the highest result in the group aged 65 years or older [Life satisfaction: coef. = 0.80 (95% CI: 0.66; 0.93), p < 0.0001; Mental wellbeing: coef. = 0.77 (95% CI: 0.67; 0.88), p < 0.0001]. Non-Western participants scored lower in both domains compared to those with a Danish background. Regarding marital status, the unmarried and divorced/separated groups had the lowest scores, with a stronger association for life satisfaction. Higher education levels were positively associated with both outcomes. Compared to being employed, all other employment statuses reported lower scores. Difficulty in paying bills every month was the strongest determinant of life satisfaction and mental wellbeing, with a negative association that intensified with frequency. Those facing monthly financial issues had the lowest scores for life satisfaction [coef. = −2.25 (95% CI: −2.49; −2.01), p < 0.0001] and mental wellbeing [coef. = −1.46 (95% CI: −1.65; −1.27), p < 0.0001]. Life satisfaction was more affected by financial difficulties than mental wellbeing. More detailed results are available in the online Supplementary Table S3.

#### Health, Health Behaviour, and Social Relations

**FIGURE 3:**
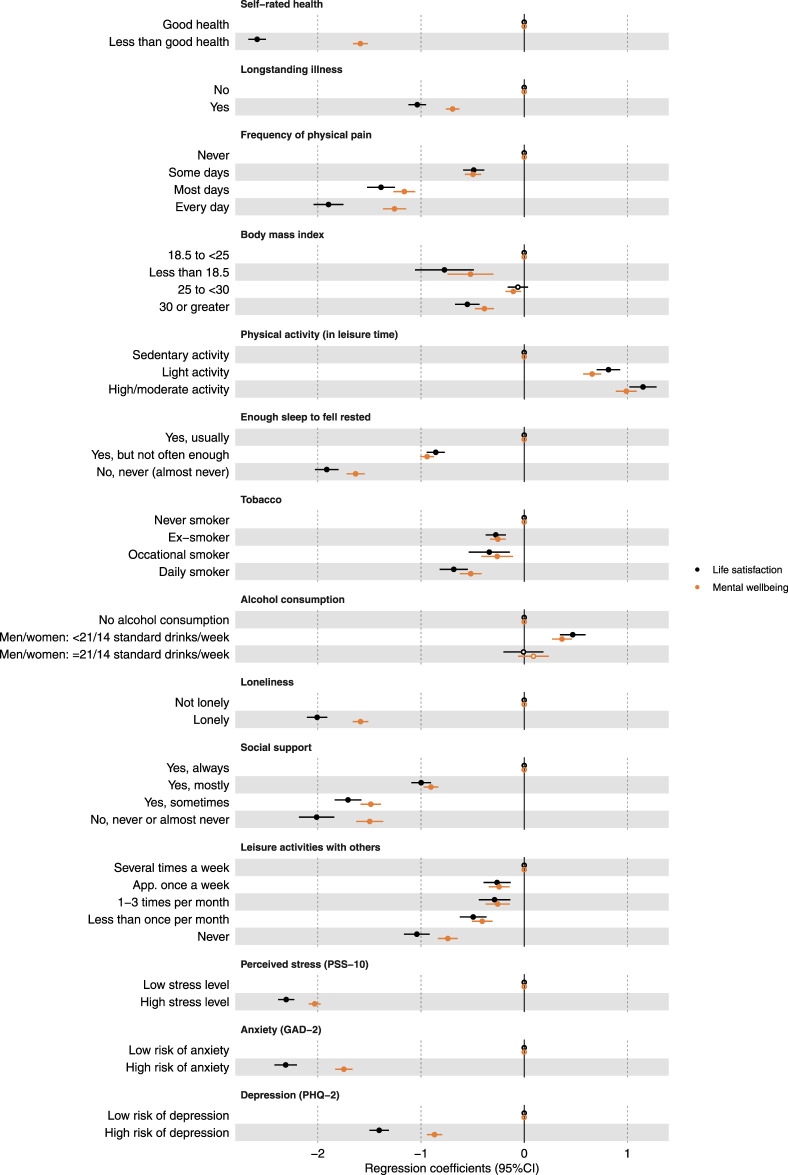
Presenting associations between life satisfaction or mental wellbeing and health, health behaviour, and social relations factors. All analyses are adjusted for sex and age and incorporating weights to account for the sampling design. Closed circles represent associations with a p-value <0.05, while open dots indicate associations with a p-value ≥0.05 (The Danish National Health Survey, Denmark, 2023).

Associations between measures of health, health behaviour, and social relations and the two outcomes are shown in Figure 3. Several strong negative determinants were identified: poor self-rated health, loneliness, high stress levels (PSS-10), and high anxiety risk (GAD-2). Additionally, longstanding illness and high risk of depression (PHQ-2) also showed negative associations, though to a lesser extent. Life satisfaction was more strongly associated with these factors than mental wellbeing. The frequency of physical pain, inadequate sleep, and lack of social support all showed a negative association, which intensified with increased pain, insufficient sleep, and absence of support. Notably, at the highest levels of these determinants, there was a notable difference between life satisfaction and mental wellbeing, with the largest regression coefficients observed for life satisfaction. A similar, though weaker, tendency was observed regarding leisure activities with others, with the strongest negative association among participants who reported never joining such activities. Regarding physical activity there was a positive association strengthening with increased activity, and the effect was similar for both outcomes. Moderate results were observed for body mass index, smoking, and alcohol consumption. Detailed results are available in the online Supplementary Table S4.

## Discussion

This study aimed to provide insights into the understanding of evaluative and mental wellbeing in a Danish context, using a sample of 10,196 adults who participated in the DNHS-2023 [15]. The correlation between the two wellbeing outcomes was found to be strong. Associations between sociodemographic factors and the scores of life satisfaction and mental wellbeing were rather similar for sex, age, ethnic background, level of education, employment status, body mass index, use of tobacco, and alcohol consumption. The scores differed regarding marital status, difficulty paying bills, self-rated health, longstanding illness, physical activity, loneliness, perceived stress level, level of anxiety, and risk of depression. The results were less clear regarding frequency of physical pain, enough sleep to feel rested, social support, and leisure activity with others, with the results being similar for some categories while others showed great differences.

The observed correlation underscores the relevance of this study, indicating that while life satisfaction and mental wellbeing are related, they are at the same time distinct constructs, each capturing unique aspects of an individual’s experiences. The consistency across the non-parametric (Spearman’s rho: ρ = 0.64) and the parametric [Pearson’s correlation: r = 0.62 (95% CI: 0.60; 0.63)] approaches suggests that the observed relationship is robust and not significantly influenced by the underlying assumptions of each method. A strong correlation implies a clear, albeit not perfect, linear relationship between the variables. It is not too strong to jeopardize discriminant validity or imply redundancy. This finding enhances the reliability of the detected correlation and supports the validity of the results, regardless of the statistical approach employed. However, it is crucial to recognize that these results may not be universally applicable to other wellbeing outcomes.

When interpreting the results within sociodemographic groups, it is evident that different wellbeing outcomes should not automatically be assumed to have the same effects on all groups. Certain factors exhibited a comparable impact on both life satisfaction and mental wellbeing (sex, age, ethnic background, level of education, employment status, body mass index, use of tobacco, and alcohol consumption), suggesting that these factors play a consistent role in shaping overall wellbeing. Conversely, other factors demonstrated divergent effects on life satisfaction and mental wellbeing (marital status, difficulty paying bills, self-rated health, longstanding illness, physical activity, loneliness, perceived stress level, level of anxiety, and risk of depression), resulting in varying impacts on the two measures. These discrepancies highlight the complexity of wellbeing and the need for a nuanced approach when addressing these factors in different contexts. Furthermore, some factors were less conclusive (frequency of physical pain, enough sleep to feel rested, social support, and leisure activity with others). While some categories showed similar effects on life satisfaction and mental wellbeing, others revealed significant differences. The inconsistencies may be due to the categorization within the groups, as the general pattern in these cases showed an increasing difference between the two measures with increasing or decreasing frequency depending on the factor in question. For example, regarding the frequency of physical pain (reference category: “never”), the results were almost identical for “some days,” less similar for “most days,” and completely different for “every day.”

It is noteworthy that education is more strongly associated with mental wellbeing, while financial problems are more strongly associated with life satisfaction. This suggests that there are several dimensions in relation to education with particular significance for mental wellbeing, whereas it is a typical finding that life satisfaction and income are closely linked [36, 37].

Overall, this variability in impact across sociodemographic groups indicates that further research is needed to fully understand the influence and patterns of these factors on evaluative and mental wellbeing. Understanding the associations between the two outcomes and other health measures will provide a deeper understanding of what aspects of wellbeing the different measures capture and represent, which is central in relation to the interpretation of results. In addition, this is important from a psychometric perspective ensuring that measures are valid and meaningful across diverse populations.

From a public health perspective — and in line with the Health Promotion Model — these insights are critical for designing interventions that not only address individual-level determinants of wellbeing (e.g., self-efficacy) but also consider broader social and environmental contexts. This approach supports the development of more holistic, equitable, and effective strategies to enhance population wellbeing and reduce health disparities. Practical interventions aligned with the Health Promotion Model include school-based programs that build emotional resilience and self-efficacy in children, and workplace initiatives that promote mental wellbeing through mindfulness, flexible schedules, and peer support. These interventions target individual-level factors such as perceived control and behavioural confidence, which are central to sustaining positive health behaviours [38–41].

At the community and systems level, social prescribing connects individuals to meaningful non-clinical activities like volunteering or nature-based programs, while urban design strategies — such as creating green spaces and walkable neighbourhoods — enhance environmental support for wellbeing. Together, these approaches address both personal and contextual determinants of health, enabling more equitable and holistic improvements in mental wellbeing and life satisfaction [38–41].

As this is the first study of its kind in Denmark, it is challenging to compare these results directly with previous findings. This emphasizes the need for further research to replicate these results and to explore the underlying mechanisms of the relationship between evaluative and mental wellbeing.

For research practice, these findings have important implications. Firstly, the study supports the use of multiple statistical methods to validate the robustness of results, which is crucial for ensuring consistent and generalizable conclusions. Secondly, it provides evidence on the discriminant validity of the two measures (i.e., partially different predictors), thereby highlighting the importance of including life satisfaction as a key variable in mental health research. This could lead to a more comprehensive understanding of mental health challenges and contribute to the development of more effective, evidence-based interventions that target both life satisfaction and mental wellbeing as measurable and modifiable factors.

The findings of this study should be seen in the light of its strengths and limitations. One of the main strengths of this study is its large sample size and representativeness of the general population. Further, the questionnaire covers a wide variety of topics not included in official statistical registers — especially regarding different measures of mental health.

However, the low response rate is a concern, as the generalizability of the collected data to the target population may be compromised. To account for these challenges, information on respondents and non-respondents was obtained from official statistical registers and analysed in the original study [15]. To statistically adjust for differential non-response, calibrated weights were applied. However, calibrated weight cannot completely upweight responses from groups that have not responded in the first place, such as residents in Denmark without Danish language skills, who are excluded because the questionnaire is available only in Danish. Further, calibrated weights may induce some standard error, reducing the precision of the estimates, and complicate analyses of the data [42]. Another general limitation of this study is the cross-sectional design, which does not allow for conclusions to be drawn on the direction of causality. Finally, the use of self-reported data may introduce reporting bias due to social desirability or inaccurate self-assessment.

### Conclusion

This study emphasizes the importance of considering a broad range of sociodemographic factors when assessing life satisfaction and mental wellbeing. The observed impact of these factors calls for nuanced public health strategies tailored to diverse needs of different population groups. These results support the development of holistic health programmes that reflect the complex interplay between physical and mental health.

These insights also have clinical implications for designing inclusive and effective interventions aimed at promoting overall wellbeing and health equity. Future research should continue to explore these dynamics to deepen our understanding of wellbeing in diverse populations and to inform, evidence-based policies and practices and ensure that interventions are both equitable and impactful across diverse populations.
