# Supplementary material for: Determinants of Life Satisfaction and Mental Wellbeing in the Danish General Population: Shared and Distinct Associations
Source: Int J Public Health. 2025 Sep 12;70:1608531. doi: 10.3389/ijph.2025.1608531 (PMC12463736; doi:10.3389/ijph.2025.1608531)
Supplement: Supplementary file 1 [file Supplementaryfile1.docx]

### Online supplemental

Table 3: Presenting associations (regression coefficients and 95% confidence interval) between life satisfaction or mental well-being and sociodemographic factors. All analyses are adjusted for sex and age and incorporating weights to account for the sampling design.

|  |  | Life satisfaction |  | SWEMWBS-10 |  | n (%) |
| --- | --- | --- | --- | --- | --- | --- |
|  |  | Coef. (95% CI) | p-value | Coef. (95% CI) | p-value |  |
| Sex | |  |  |  |  |  |
|  | Men | 0 |  | 0 |  | 4481 (44.0) |
|  | Women | -0.08 (-0.16;0.01) | 0.0668 | -0.15 (-0.21;-0.08) | <0.0001 | 5715 (56.0) |
| Age | |  |  |  |  |  |
|  | 16-24 years | 0 |  | 0 |  | 913 (9.0) |
|  | 25-44 years | 0.08 (-0.05;0.22) | 0.2281 | 0.09 (-0.02;0.20) | 0.0964 | 2120 (20.8) |
|  | 45-64 years | 0.39 (0.26;0.52) | <0.0001 | 0.46 (0.35;0.56) | <0.0001 | 3517 (34.5) |
|  | 65 years or older | 0.80 (0.66;0.93) | <0.0001 | 0.77 (0.67;0.88) | <0.0001 | 3646 (35.8) |
| Ethnic background | |  |  |  |  |  |
|  | Danish | 0 |  | 0 |  | 9407 (92.3) |
|  | Western | -0.12 (-0.28;0.05) | 0.1614 | -0.20 (-0.32;-0.07) | 0.0027 | 369 (3.6) |
|  | Non-Western | -0.22 (-0.36;-0.07) | 0.0035 | -0.44 (-0.56;-0.32) | <0.0001 | 420 (4.1) |
| Marital status | |  |  |  |  |  |
|  | Married/cohabiting | 0 |  | 0 |  | 5576 (54.7) |
|  | Divorced/separated | -0.47 (-0.60;-0.33) | <0.0001 | -0.18 (-0.29;-0.08) | 0.0005 | 1180 (11.6) |
|  | Widowed | -0.22 (-0.40;-0.04) | 0.0168 | -0.04 (-0.19;-0.10) | 0.5396 | 699 (6.9) |
|  | Unmarried | -0.74 (-0.85;-0.63) | <0.0001 | -0.47 (-0.56;-0.38) | <0.0001 | 2741 (26.9) |
| Education level | |  |  |  |  |  |
|  | Basic school | 0 |  | 0 |  | 849 (10.5) |
|  | Upper secondary or vocational school | 0.33 (0.19;0.48) | <0.0001 | 0.34 (0.22;0.45) | <0.0001 | 2769 (34.2) |
|  | Higher education | 0.44 (0.30;0.58) | <0.0001 | 0.51 (0.40;0.62) | <0.0001 | 4469 (55.3) |
| Employment | |  |  |  |  |  |
|  | Employed | 0 |  | 0 |  | 4370 (43.7) |
|  | Unemployed | -0.93 (-1.19;-0.67) | <0.0001 | -0.78 (-1.01;-0.56) | <0.0001 | 192 (1.9) |
|  | Pensioner/early retirement | -0.84 (-1.01;-0.67) | <0.0001 | -0.85 (-1.00;-0.70) | <0.0001 | 4274 (42.7) |
|  | Other | -0.96 (-1.10;-0.82) | <0.0001 | -0.67 (-0.78;-0.56) | <0.0001 | 1171 (11.7) |
| Difficulty paying bills | |  |  |  |  |  |
|  | Never | 0 |  | 0 |  | 8014 (87.1) |
|  | Some few months | -0.85 (-0.98;-0.72) | <0.0001 | -0.70 (-0.80;-0.60) | <0.0001 | 817 (8.9) |
|  | App. half of the months in a year | -1.82 (-2.07;-1.56) | <0.0001 | -1.14 (-1.34;-0.94) | <0.0001 | 172 (1.9) |
|  | Every month | -2.25 (-2.49;-2.01) | <0.0001 | -1.46 (-1.65;-1.27) | <0.0001 | 195 (2.1) |

Table 4: Presenting associations (regression coefficients and 95% confidence interval) between life satisfaction or mental well-being and health, health behaviour, and social relations factors. All analyses are adjusted for sex and age and incorporating weights to account for the sampling design.

|  |  | Life satisfaction |  | SWEMWBS-10 |  | n (%) |
| --- | --- | --- | --- | --- | --- | --- |
|  |  | Coef. (95% CI) | p-value | Coef. (95% CI) | p-value |  |
| Health (self-rated) | |  |  |  |  |  |
|  | Good health | 0 |  | 0 |  | 7946 (77.9) |
|  | Less than good health | -2.59 (-2.67;-2.50) | <0.0001 | -1.59 (-1.66;-1.52) | <0.0001 | 2184 (21.4) |
| Longstanding illness | |  |  |  |  |  |
|  | No | 0 |  | 0 |  | 5398 (57.1) |
|  | Yes | -1.04 (-1.12;-0.95) | <0.0001 | -0.69 (-0.76;-0.63) | <0.0001 | 4061 (42.9) |
| Frequency of physical pain | |  |  |  |  |  |
|  | Never | 0 |  | 0 |  | 2039 (21.7) |
|  | Some days | -0.49 (-0.59;-0.39) | <0.0001 | -0.50 (-0.58;-0.42) | <0.0001 | 4665 (46.7) |
|  | Most days | -1.39 (-1.52;-1.25) | <0.0001 | -1.16 (-1.67;-1.06) | <0.0001 | 1421 (15.2) |
|  | Every day | -1.90 (-2.04;-1.75) | <0.0001 | -1.26 (-1.37;-1.14) | <0.0001 | 1254 (13.4) |
| BMI | |  |  |  |  |  |
|  | <18.5 | -0.77 (-1.06;-0.49) | <0.0001 | -0.52 (-0.74;-0.30) | <0.0001 | 203 (2.2) |
|  | 18.5 - <25.0 | 0 |  | 0 |  | 4022 (44.3) |
|  | 25.0 - <30.0 | -0.06 (-0.16;0.04) | 0.2256 | -0.11 (-0.18;-0.03) | 0.0069 | 3167 (34.9) |
|  | ≥30 | -0.55 (-0.67;-0.43) | <0.0001 | -0.39 (-0.48;-0.30) | <0.0001 | 1686 (18.6) |
| Physical activity (in leisure time) | |  |  |  |  |  |
|  | Sedentary activity | 0 |  | 0 |  | 1482 (16.2) |
|  | Light activity | 0.81 (0.70;0.93) | <0.0001 | 0.66 (0.57-0.75) | <0.0001 | 5529 (60.5) |
|  | High/moderate activity | 1.15 (1.02;1.28) | <0.0001 | 0.99 (0.89-1.09) | <0.0001 | 2128 (23.3) |
| Enough sleep | |  |  |  |  |  |
|  | Yes, usually | 0 |  | 0 |  | 5266 (53.2) |
|  | Yes, but not often enough | -0.86 (-0.94;-0.77) | <0.0001 | -0.94 (-1.01;-0.87) | <0.0001 | 3230 (32.6) |
|  | No, never (almost never) | -1.91 (-2.03;-1.80) | <0.0001 | -1.63 (-1.72;-1.55) | <0.0001 | 1410 (14.2) |
| Tobacco | |  |  |  |  |  |
|  | Never smoked | 0 |  | 0 |  | 4811 (51.4) |
|  | Ex-smoker | -0.28 (-0.37;-0.18) | <0.0001 | -0.26 (-0.33;-0.18) | <0.0001 | 3219 (34.4) |
|  | Occasional smoker | -0.34 (-0.54;-0.14) | 0.0009 | -0.26 (-0.42;-0.11) | 0.0008 | 371 (4.0) |
|  | Daily smoker | -0.68 (-0.82;-0.55) | <0.0001 | -0.52 (-0.62;-0.41) | <0.0001 | 968 (10.3) |
| Alcohol consumption | |  |  |  |  |  |
|  | No alcohol consumption | 0 |  | 0 |  | 1079 (11.9) |
|  | Men/women: <21/14 std. drinks/week | 0.47 (0.35;0.59) | <0.0001 | 0.37 (0.27;0.46) | <0.0001 | 7222 (70.8) |
|  | Men/women: ≥21/14 std. drinks/week | -0.01 (-0.20;0.19) | 0.3600 | 0.09 (-0.02;0.21) | 0.2392 | 742 (7.3) |
| Loneliness | |  |  |  |  |  |
|  | Not lonely | 0 |  | 0 |  | 7546 (82.6) |
|  | Lonely | -2.01 (-2.11;-1.91) | <0.0001 | -1.59 (-1.66;-1.51) | <0.0001 | 1587 (17.4) |
| Social support | |  |  |  |  |  |
|  | Yes, always | 0 |  | 0 |  | 5541 (54.3) |
|  | Yes, mostly | -1.00 (-1.09;-0.90) | <0.0001 | -0.90 (-0.98;-0.83) | <0.0001 | 2301 (22.6) |
|  | Yes, sometimes | -1.71 (-1.84;-1.58) | <0.0001 | -1.49 (-1.59;-1.39) | <0.0001 | 916 (9.0) |
|  | No, never or almost never | -2.01 (-2.18;-1.84) | <0.0001 | -1.50 (-1.63;-1.67) | <0.0001 | 446 (4.4) |
| Leisure activities with others | |  |  |  |  |  |
|  | Several times a week | 0 |  | 0 |  | 2114 (23.0) |
|  | App. once a week | -0.26 (-0.39;-0.13) | 0.0001 | -0.24 (-0.34;-0.14) | <0.0001 | 1920 (20.9) |
|  | 1-3 times per month | -0.29 (-0.44;-0.13) | 0.0002 | -0.26 (-0.38;-0.14) | <0.0001 | 1109 (12.1) |
|  | Less than once per month | -0.49 (-0.62;-0.37) | <0.0001 | -0.41 (-0.51;-0.31) | <0.0001 | 1892 (20.6) |
|  | Never | -1.04 (-1.17;-0.91) | <0.0001 | -0.74 (-0.84;-0.64) | <0.0001 | 2160 (23.5) |
| Perceived stress (PSS-10) | |  |  |  |  |  |
|  | Medium/low stress level (<18) | 0 |  | 0 |  | 6806 (71.8) |
|  | High stress level (≥18) | -2.31 (-2.39;-2.23) | <0.0001 | -2.03 (-2.09;-2.00) | <0.0001 | 2668 (28.2) |
| Anxiety (GAD-2) | |  |  |  |  |  |
|  | Low risk of anxiety (<3) | 0 |  | 0 |  | 8165 (86.8) |
|  | High risk of anxiety (≥3) | -2.31 (-2.42;-2.20) | <0.0001 | -1.75 (-1.83;-1.66) | <0.0001 | 1245 (13.2) |
| Depression (PHQ-2) | |  |  |  |  |  |
|  | Low risk of depression (<3) | 0 |  | 0 |  | 7226 (77.3) |
|  | High risk of depression (≥3) | -1.41 (-1.50;-1.31) | <0.0001 | -0.87 (-0.94;-0.79) | <0.0001 | 2124 (22.7) |
